# Switching Loss Model for SiC MOSFETs Based on Datasheet Parameters Enabling Virtual Junction Temperature Estimation

**DOI:** 10.3390/s25123605

**Published:** 2025-06-08

**Authors:** Claudio Bianchini, Mattia Vogni, Alessandro Chini, Giovanni Franceschini

**Affiliations:** Department of Engineering Enzo Ferrari, University of Modena and Reggio-Emilia, 41125 Modena, Italy; mattia.vogni@unimore.it (M.V.); alessandro.chini@unimore.it (A.C.); giovanni.franceschini@unimore.it (G.F.)

**Keywords:** SiC MOSFETs, switching losses, efficiency, measurement uncertainty, virtual junction temperature

## Abstract

SiC MOSFETs are widely employed in power converters due to their superior efficiency and reliability at high temperatures. For this reason, it is crucial to implement accurate thermal models capable of indirectly estimating the junction temperature and its fluctuations: both are caused by power losses in the device. In this framework, the evaluation of switching losses remains the most challenging task. To enable real-time monitoring of the junction temperature, this work presents the development of a virtual sensor specifically designed for SiC MOSFETs. The sensor relies on a num-analytical model (NAM), which employs only datasheet parameters and leverages electrical quantities—namely, bus voltage and current—available from sensors integrated into power converter systems. The proposed NAM is implemented in MATLAB using an iterative algorithm that accounts for the main physical phenomena involved in switching transitions. The computed energy losses are then used to thermally model the SiC MOSFETs within the PLECS environment, where a digital twin of an all-SiC board is created. Finally, the accuracy of the model is validated by comparing simulation results with experimental efficiency data obtained from a real half-bridge converter, with explicit consideration of measurement uncertainty.

## 1. Introduction

### 1.1. Motivation

Advanced modeling and simulation of electrical equipment are key issues in the development and design of high-efficiency power converters for industrial and automotive applications. The constantly growing interest in SiC-based devices is a natural result of their better merit figures compared to traditional Si-based solutions [1,2,3]. The adoption of SiC devices typically allows for a significant increase in both efficiency and power density thanks to their faster switching time and reduced conduction losses. Moreover, they can also operate at higher temperatures [4]. While on the performance side, the advantage is clear and sound, cost-wise, the adoption of SiC-based devices needs to deal with a trade-off between the final system improvements and cost. Even though the advantage on the performance side is clear and sound, the increase in power density provokes a rise in thermal stress on SiC MOSFETs, which leads to a decrease in the system MTTF (Mean Time To Failure) without appropriate monitoring of losses and device temperature. Since the junction temperature and its fluctuation are responsible for the thermal aging and failure of the transistors, it is essential to monitor this physical quantity. A virtual sensor, especially, allows for the estimation of the junction temperature both online under a real operating condition and offline if the converter/inverter behavior and load profile are known beforehand, avoiding the need for thermocouples. These sensors are also less reliable in high-end applications with bare-die components, since they cannot be placed on the devices, but close to them. Because the temperature depends on the thermal chain between the device and the ambient and on the power dissipated, a proper estimation of the transistor losses represents the first and most important step of a lifetime evaluation. Several lifetime models and procedures already exist in the literature for traditional IGBT devices [5,6,7]. Similarly, some were developed for SiC MOSFETs: the authors of [8] presented a great overview of the state of the art. Considering also that in high-power applications, an error of 1 % on the efficiency estimation could significantly impact the heat sink design and the online evaluation of the thermal status, the importance of estimating power loss in a SiC MOSFET becomes evident.

### 1.2. Overview of the Topic

In this framework, the evaluation of switching losses remains the most challenging task. Therefore, in recent years, several authors have put their efforts into the development of a model that could estimate them [9,10,11,12,13,14,15,16,17,18,19,20,21,22,23,24]. An old and well-established model such as [9] is very easy to understand and implement; however, it lacks precision due to the strong approximations made on the gate drain capacitance and on constant rise and fall times. Others such as refs. [10,11,12] are very complete but require preliminary measurements to derive the dynamic characteristics. Refs. [11,12] both take into account the short-channel and the drain-induced lowering barrier (DIBL) effects, but they require a single-pulse test to obtain the saturation characteristic. Even though this approach is formally correct, it requires a dedicated setup for parameter extraction and additional time, which may be very impactful for several companies approaching a SiC power converter design. The authors of [13] made great efforts in the analytical derivation of turn-on losses. However, even though turn-on losses are predominant, turn-off losses are not negligible. Other papers, such as [14], focused on the influence of single parameters on switching losses, charge and discharge $C_{oss}$ in this case, which is relevant for $E_{on}$ and $E_{off}$ computation but introduces a further level of complexity, which is secondary in the total $E_{tot}$ computation. Some numerical analytical models based on datasheet parameters already exist in the literature [15,16]. They either are mathematically challenging [15] or make strong assumptions on the equivalent transconductance and capacitance [16]. In both cases, it is not clear how to estimate the parasitic inductances without carrying out any measurement. These models work well with discrete SiC devices in very standard packages; in this way, these lumped parameters can be assumed from similar part numbers, but for bare-die components or non-standard packages, additional measures would be mandatory. The authors of [17] focused on reverse-recovery estimation, but their method also requires preliminary measurements. The same goes for [18], who adapted the model for a specific condition (quasi-zero turn-off losses). Moreover, their procedure also demands previous measurements. Ref. [19] made strong assumptions on temperature independency and also required the computation of some dynamic characteristics. Refs. [10,11,12,13,14,15,16,17,18,19] are all num-analytical models (NAMs), such as the one presented in this work; in addition, full-analytical models (FAMs) exist [20,21,22,23,24]. Refs. [20,21,22] studied linearized waveforms and, especially the first two, also made an assumption of constant transconductance. The most complete FAM in the literature is the one derived by Hu and Biela in [23,24]. However, its mathematical complexity is extremely challenging, and even though they provide good accuracy, they make step-wise approximations and a temperature independency assumption of the $E_{on}$ and $E_{off}$ losses. These assumptions become relevant as the switching frequency increases. In this paper, a new NAM model is therefore presented, entirely based on datasheet parameters, to estimate the switching losses of an application with all-SiC, SiC MOSFETs, and SiC Schottky diodes in anti-parallel. This model is based on an iterative method, which can be solved in any commonly used language: C++, MATLAB, Python, etc. This model considers all the possible information provided by the manufacturer: non-linear capacitance, channel modulation mode, and dependency of $t_{ri}$ and $t_{fi}$ on the load current. Nevertheless, this approach aims to be easily implemented by any company or research group, following the trend of a more efficient and widespread electric vehicle drive design and of the development of renewable energy systems with better performances. Moreover, none of the aforementioned models were validated considering the efficiency computation and the measurement uncertainty, which makes this work innovative in the literature perspective.

### 1.3. Article Structure

In Section 2, the SiC MOSFET switching loss model is explained in comparison with a simplified reference model [9], an analytical model of similar complexity [16], and a FAM model [24]. At the end of the section, its universality is discussed. Proceeding to Section 3, the setup for the experiments is presented. In Section 4, a digression on the expanded uncertainty evaluation is carried out: this is a needed premise to compare the measured efficiency with the one obtained in the simulation. In the same section, the results are summarized and compared with the aforementioned models. Finally, some conclusions and future development hints are given in Section 5 and Section 6.

## 2. SiC MOSFET Switching Loss Evaluation

### 2.1. Introduction to the Analysis

In this section, after presenting the well-known half-bridge architecture and its main parasitic components, a brief overview of the state of the art of SiC switching loss estimation is presented. Then, the proposed model is detailed and shown in comparison with three existing ones: the simplified reference model found in [9], a NAM of similar complexity in [16], and a FAM in [24]. Afterwards, a paragraph is dedicated to the universality of the model, and at the end of the section, a subsection addresses the junction temperature estimation based on the proposed model.

### 2.2. Half-Bridge Architecture and Lumped Parasitic Parameters

**Figure 1:**
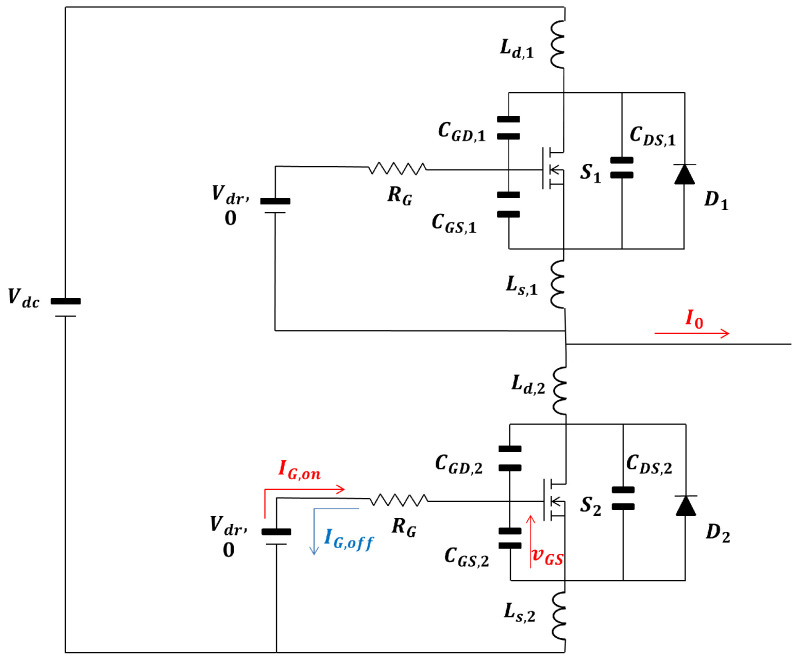
SiC MOSFET electric model with parasitic inductance.

The architecture under study consists of a half-bridge with two SiC MOSFETs and two SiC Schottky diodes in anti-parallel. The same architecture is tested in Section 3. The electric model including all parasitic components is drawn in Figure 1 and is common throughout the section. Regarding the parameters, $R_{G}$ is the sum of the external and internal gate resistance; further, in this section, $R_{G,on}$, the total gate resistance during turn-on, and $R_{G,off}$, the total gate resistance during turn-off, are distinguished; $L_{s}$ and $L_{d}$ are the parasitic inductance of the source and drain, respectively; $C_{GS}$, $C_{GD}$, and $C_{DS}$ are the capacitance of the gate source, gate drain, and drain source, respectively; $V_{DC}$ is the DC bus supply voltage; and $V_{dr}$ is the high-level voltage gate supply, with off-state 0 V applied to the control pins.

### 2.3. Overview of SiC Switching Loss Models

As mentioned in the Introduction, several authors tried to model switching losses in recent years; some of them also proved good compliance with the experimental tests, which had always consisted of a double-pulse test (DPT). However, each of them lacks either accuracy or results in excessive complexity. Moreover, most of them require some preliminary tests for parameter extraction. The authors of this work, instead, believe in ease of application of the model with the best possible accuracy. Here, a brief summary of each model problem is provided, and the synthesis can be found in Table 1. Ref. [9] is better detailed later on in this section, since it will be used as one of the benchmarks in Section 4: it consists of a well-established but very raw model. $C_{GD}$ is approximated as a step function and current rise and fall times are considered independent from the load current. Ref. [10] presented a good summary of several factors influencing the switching loss mechanism; however, it demands the measurement of dynamic characteristics of the MOSFET parasitic capacitances. Moreover, several variables, which are changed on purpose in the analysis, are normally parameters of the already existing power board, such as the loop inductance $L_{loop}$. Other parameters are difficult to estimate: the parasitic inductances $L_{s}$ and $L_{d}$ cannot be easily computed; usually a DPT is required or a very accurate LTSpice model, provided by the manufacturer. In most cases, this is not possible without building a dedicated setup. Furthermore, an error of 5% on the total energy losses is significant: especially at high switching frequencies, it leads to significant deviation on the efficiency estimation. Ref. [11] shows better accuracy but maintains the same problem of the dynamic characterization of $Q_{gd}$, the gate drain charge, and of the transfer characteristic of the device, resulting in time and practical effort. Ref. [12] is very accurate but presents the same problem of previous characterization. In [13], great insights on the energy turn-on loss computation are given, but even if they represent the predominant part, the turn-off losses are not negligible. On the contrary, Ref. [14] focuses on the charge and discharge of the output capacitance $C_{oss}$, which is a real physical phenomenon but does not allow for better estimation of the total losses, since the energy absorbed in one switching transient, $E_{oss}$, is then returned in the other: aiming to estimate the efficiency; this sophistication can be neglected. The authors of [15,16], which is detailed later on, provided a NAM based on datasheet parameters, even though the estimation of $L_{s}$ and $L_{d}$ and its difficulty are not well delved into. The first one showed a great accuracy but high mathematically complexity. Ref. [16] is lacking a bit in accuracy, even though it is more understandable. Ref. [17], on the contrary, requires preliminary tests to extract the lumped parameters, even though it is accurate. Ref. [18] gave interesting insights into the switching losses in the quasi-ZTL (zero turn-off losses) condition but remained very specific and needs preliminary tests for the dynamic $C_{GD}$ curve extraction. The exact same problem can be found in [19], where the authors made an assumption of temperature independency, as well. Full analytical models such as [20,21,22] supposed linearized waveforms. These assumptions, added to a constant transconductance supposition, allow for faster evaluation but reduce the models’ accuracy. Finally, Hu and Biela developed a FAM in [23] and refined it in [24], which is quite accurate and based on datasheet parameters, even though it makes strong assumptions such as temperature independency and equivalent capacitance approximation. To reduce the computational effort, which is a goal for models of this kind, it solves all the differential equations in a closed form, leading to a very challenging and almost prohibitive complexity. Also, this model is briefly described later on in this section.

### 2.4. Simplified Reference Model

First, the simple model proposed by [9], based on datasheet parameters, is taken into account. This model gives a rough estimation of switching losses: in fact, it assumes a linear approximation for both voltage and current during turn-on and turn-off transients. During the current rise time $t_{ri}$, the gate source voltage $v_{GS}$ increases linearly from the threshold voltage $V_{th}$, to the Miller plateau voltage $V_{mil}$, which is considered constant and equal to the typical value reported in the datasheet. The same happens during $t_{ri}$ in the turn-off transient: $v_{GS}$ decreases linearly from $V_{mil}$ to $V_{th}$. The model assumes that both the rising time of the current $t_{ri}$ and the falling time of the current $t_{fi}$ are constant and equal to their typical values, which can be found in the datasheet. The voltage fall time, during the turn-on transient $t_{fu}$ is the time required to discharge $C_{GD}$. The discharging process starts when the drain source voltage $v_{DS}$ equals the supply voltage $V_{DC}$ and ends when the MOSFET is conducting, i.e., when $v_{DS}$ reaches the drain source voltage of the MOSFET in the on-state $V_{DS,on}=R_{DS,on}\cdot i_{D}$, where $R_{DS,on}$ is the device on resistance. At the beginning of $t_{fu}$, the voltage across $C_{GD}$ is $V_{DC}-V_{mil}$ and, at the end, is $V_{DS,on}-V_{mil}$; therefore, it is possible to consider the discharging of the capacitance at the constant current $I_{G,on}$ provided by the gate driver. This statement is not an assumption, since $v_{GS}$ is clamped to $V_{mil}$ throughout the period of time. Time $t_{fu}$ could be estimated as in (1).

(1) $$t_{fu}=\frac{\Delta V\cdot C_{GD}}{I_{G,on}}=\left(V_{DC}-V_{DS,on}\right)R_{G,on}\cdot \frac{C_{GD}}{V_{dr}-V_{mil}}$$

where $V_{dr}$ is the voltage applied by the gate driver. One of the main issues is the value assumed by $C_{GD}$ or $C_{rss}$, reverse transfer capacitance. Since this capacitance is dependent on the drain source voltage $v_{DS}$, the authors of [9] suggest a zero-hold interpolation based on two points: the first one at $V_{DS,on}$ and the second one at $V_{DC}$ for the $(C_{rss}$-$v_{DS})$ curve that is found in the datasheet. A falling time is correlated with both $C_{rss}$ values. The mean value between the two falling times is $t_{fu}$. Therefore, the switch-on losses $E_{on}$ can be calculated as the area of a triangle, as can be seen in (2).

(2) $$E_{on}=\frac{1}{2}V_{DC}I_{0}\cdot \left(t_{ri}+t_{fu}\right)$$

where $I_{0}$ is the load current. A very similar approach is employed to evaluate the voltage rise time $t_{ru}$ and, therefore, to calculate the turn-off losses $E_{off}$, with two differences: the voltage applied by the gate driver is 0 *V* and the total gate resistance $R_{G,off}$ is generally different from $R_{G,on}$.

As can be seen in Section 4, the Infineon model leads to a significant overestimation of the switching losses. Although very simple, it lacks of precision. The following subsection analyzes the num-analytical model of Christen and Biela [16] in brief.

### 2.5. Existing Analytical Model of Similar Complexity

In this subsection, the steps employed by the authors of [16] to evaluate $E_{on}$ are briefly resumed; $E_{off}$ are calculated in a similar way. Firstly, the input capacitance $C_{iss}=C_{GS}+C_{GD}$, the output capacitance $C_{oss}=C_{DS}+C_{GD}$, and the reverse-transfer capacitance $C_{rss}=C_{GD}$ are considered fixed at an equivalent value, calculated through the integral of each respective curve. The capacitances are then derived from algebraic operations of the three above. From $C_{oss,eq}$, an equivalent value is derived for the charge stored in $C_{oss}$, $Q_{oss,eq}$. Because $C_{rss}$ plays an important role in the voltage switching transients, it is believed that this approximation is the main cause of the deviation with respect to the experimental results, which can be appreciated in Section 4. Then, a second-order equation, if parasitic inductances $L_{s}$ and $L_{d}$ are considered, or a first-order equation, if not, is solved through a numerical method, for example, the Newton–Raphson method, in order to obtain a value for $I_{oss}$. $I_{oss}$ is the current needed to charge $C_{oss}$ during the current rise. A value for the transconductance $g_{m}$ is then found [25].

Consider that $I_{oss}$ is negative during turn-on and positive during turn-off, which means that the actual current flowing in the channel during the voltage drop is greater than the load current and vice versa during the voltage rise [26]. Thus, the current rise time $t_{ri}$ and the voltage fall time $t_{fu}$ are calculated through (3).

(3) $$\left\{\begin{array}{c} t_{fu}=-\frac{Q_{oss,eq}}{I_{oss}}\\ t_{ri}=-\ln\left(1-\frac{Io}{g_{m}\left(V_{dr}-V_{th}\right)}\right)\left(C_{GS}R_{G,on}+L_{s}g_{m}\right) \end{array}\right.$$

Finally, if the reverse-recovery phenomenon is neglected, $E_{on}$ is calculated as in (4). A similar approach is followed for $E_{off}$ losses.

(4) $$E_{on}=\frac{1}{2}t_{ri}V_{DS,0}I_{0}+\frac{1}{2}t_{fu}V_{DS,0}\left(I_{0}-2I_{oss}\right)$$

where $V_{DS,0}=V_{DC}-L_{d}\left(I_{0}/t_{ri}\right)$ if parasitic inductances are considered; here, $L_{d}=0$. This model is implemented in MATLAB, similarly to the one proposed in this paper.

### 2.6. Fully Analytical Model

A merit of a fully analytical model is its computational time, which is reduced significantly, usually at the expense of its accuracy. However, very recently, a fast and accurate FAM was developed by Hu and Biela [23,24] at the expense of its complexity. Solving the equations of the switching transients in closed form is mathematically challenging, and even if the algorithm is provided, the implementation remains difficult Moreover, its performances are inferior, even if slightly, to the proposed model. Ref. [24] divides the turn-on and turn-off switching intervals into six parts, and for each, it computes a contribution to the total loss. The model was always implemented in MATLAB, as proposed in the next section.

### 2.7. Proposed Analytical Model

#### 2.7.1. Overview and Assumptions of the Proposed Analytical Model

This section delves into the proposed model, starting from its assumptions, which are only the necessary hypotheses to implement a fully datasheet-based model. Then, its merits will be manifested. Here is a list of the three assumptions.

1. Parasitic inductances $L_{d}$ and $L_{s}$ are neglected. Because bare-die components are employed, typical inductance values of a standard package cannot be used. The overall inductance of the half-bridge could be estimated, but its distribution in every parasitic inductance is rather challenging. Considering that the PCB layout is achieved while trying to minimize these inductances as much as possible and that the highest switching frequency at which the half-bridge is tested is 80 kHz, it is assumed that they do not play a significant role.
2. Reverse-recovery losses are neglected. SiC Schottky diodes are placed in anti-parallel to the SiC MOSFETs, and since they manifest a very low reverse-recovery peak current $I_{rr}$, the assumption is acceptable. The omission of reverse-recovery losses is compelling if SiC Schottky diodes are employed in anti-parallel to SiC MOSFETs. If, instead, body diodes of the SiC MOSFET are used, these losses should be considered. In this case, refs. [16,17] propose an effective way to compute the reverse-recovery losses. Particularly, in [16], a set of formulae is derived from the physical model presented in [27], which could be numerically solved with the Newton–Raphson method.
3. Drain-induced Barrier Lowering (DIBL) and the short channel effect are neglected. In the switching process, especially during current rise or fall, depending on which transient, turn-on or turn-off, is considered, the MOSFET operates in the saturation region. During this period of time, when powered by high DC bus voltages, the device experiences the DIBL effect, resulting in an increase in the channel current for a given $v_{GS}$ and in a reduction in the threshold voltage $V_{th}$. The complete expression of the channel current would be described by (5), as stated in [12]. With respect to the $i_{d}$-$v_{GS}$ characteristic provided by the manufacturers at low and fixed $v_{DS}$, the real characteristic at higher bus voltages is shifted to the left and expresses a higher slope. Thus, the DIBL effect neglect implies an overestimation of the losses during current rise and fall. However, the estimation of λ requires an experimental characterization of the device, especially a single-pulse test circuit [11], which would be out of the scope of this paper. The initial estimation of λ as described in [28] is not possible for most of the SiC MOSFET part numbers, and the relation between $V_{th}$ and $v_{DS}$ cannot be derived from the datasheet. Therefore, the effect is neglected.
  (5) $$i_{ch}=\frac{K_{p}}{2}\cdot {\left(v_{GS}-V_{th}\right)}^{2}\cdot \left(1+\lambda v_{DS}\right)$$
  where $i_{ch}$ is the channel current, $K_{p}$ is the transconductance coefficient, and λ is the short-channel coefficient.

Before proceeding, it should be clarified that the channel current is considered equal to the load current during $t_{fu}$ and $t_{ru}$: $i_{ch}=i_{D}$. The actual current flowing in the channel during turn-on, when the voltage drops, is greater than the load current because an additional current must be applied to discharge $C_{oss}=C_{GD}+C_{DS}$. The opposite happens during turn-off. As explained in [26], the actual $E_{on}$ losses are greater than the ones measured throughout the use of a voltage and a current probe; in contrast, the measured $E_{off}$ losses are lower than the measured ones. However, if the aim is to evaluate the efficiency, this deviation is no longer significant since $E_{oss}$ is stored but then completely released throughout the switching period, which allows one to simplify the model.

Apart from the aforementioned assumptions, the model aims to maintain the highest possible accuracy, taking into account the main aspects of the switching process. Differently from [10,11,12,13,14,15,17], it does not require prior measurements. Similarly to [16], it employs an iterative method, and consequently, it avoids the direct resolution of differential equations. However, a step-by-step with feedback updating of the main variables is chosen instead of making strong assumptions on the capacitances and on the transfer characteristic. This allows for obtaining, on average, a better accuracy than that in [24], while maintaining good understandability, avoiding closed-form expressions. The proposed model chooses to compute the main switching times $t_{ri}$, $t_{fi}$, $t_{fu}$, and $t_{ru}$ with a step-by-step algorithm. A merit of the model is also to be efficiency-oriented, since it omits the calculation of $E_{oss}$.

#### 2.7.2. $E_{on}$ Calculation

About the $E_{on}$ switching losses, two contributions are distinguished: one due to the current raising and the other to the voltage falling. These processes are considered separately, as in the simplified model. Before the current rises, when $v_{GS}<V_{th}$, the channel current is negligible and therefore the power loss contribution of this time period. After the voltage drop $v_{GS}$ goes from $V_{mil}$ to $V_{dr}$ but $v_{DS}=V_{DS,on}$, which is very close to zero, this contribution to power loss is also negligible. Since during $t_{ri}$, it is true that $v_{DS}>v_{GS}-V_{th}$, the condition of channel modulation is considered true. The same formula explained in [25] and employed in [16] can be used.

(6) $$i_{ch}=k_{1}\cdot {\left(v_{GS}-V_{th}\right)}^{x}+k_{2}$$

where $k_{1},\;k_{2}$ and *x* are three coefficients, which can be found from a fitting of the $v_{GS}-i_{D}$ curve that is found in the datasheet. For the tested part number, these values can be extracted for 25 °C and 150 °C.

(7) $$\left\{\begin{array}{c} k_{1,25^{\circ }C}=0.0638\\ k_{2,25^{\circ }C}=-0.06898\\ x_{25^{\circ }C}=3.20758 \end{array}\right.\left\{\begin{array}{c} k_{1,150^{\circ }C}=0.1935\\ k_{2,150^{\circ }C}=-0.08727\\ x_{150^{\circ }C}=2.86988 \end{array}\right.$$

$v_{GS}$ varies during $t_{ri}$, and $v_{GS}$ defines the trend of $i_{ch}$, which is equal to $i_{D}$ during this time period. The two main issues in the evaluation of the switching loss contribution of this time period are the non-linear trend of $i_{ch}$ during $t_{ri}$ and the dependence of $t_{ri}$ by the drain current itself. These difficulties in the power loss evaluation are solved by employing a feedback algorithm. After defining a time-step dt=0.01 ns, $v_{GS}$ is initialized and $V_{mil}$ is derived from (6) at load current $I_{0}$. The $dv_{GS}/dt$ expression is also defined by hypothesizing that $v_{GS}$ changes linearly during $t_{ri}$.

(8) $$\left\{\begin{array}{c} v_{GS}=V_{th}\\ V_{mil}=\sqrt[{x}]{\frac{I_{0}-k_{2}}{k_{1}}}+V_{th}\\ \frac{dv_{GS}}{dt}=\frac{V_{mil}-V_{th}}{t_{ri}} \end{array}\right.$$

where $I_{0}$ is the load-considered current; $V_{th}$, *x*, $k_{1}$ and $k_{2}$ are constant and assume different values if $T_{j}=25$ °C or $T_{j}=150$ °C; and $t_{ri}$ is initialized at the lowest available value based on the datasheet curve. A good amount of points can be extracted from the $\left(t_{ri}-i_{D}\right)$ curve in the datasheet, and then, a zero-hold interpolation is considered for this curve, i.e., the value of $t_{ri}$ is constant in between the two following samples. The number of points, if considerable, increases the precision of the model: in this case, 100 are taken in correspondence with the 100 possible load currents that were considered to build the model (from 0 A to 50 A with a step of 0.5 A). After that, a *while* cycle starts and continues until either $v_{GS}<V_{mil}$ or $i_{ch}\;<\;I_{0}$ becomes false. Inside the cycle, if the new value of $i_{ch}$ surpasses the mean value of $i_{D}$ between one sample and the following one, both $t_{ri}$ and $dv_{GS}/dt$ are updated (9). At each iteration of the *while* cycle, whether the above mentioned inequality is true or not, $v_{GS}$ and $i_{ch}$ are calculated: see (10).

(9) $$\left\{\begin{array}{c} t_{ri}\left(i-1\right)=t_{ri}\left(i\right)\\ \frac{dv_{GS}}{dt}\left(i\right)=\frac{V_{mil}-V_{th}}{t_{ri}\left(i\right)} \end{array}\right.$$

(10) $$\left\{\begin{array}{c} v_{GS}\left(i\right)=v_{GS}\left(i-1\right)+\frac{dv_{GS}}{dt}\left(i\right)\cdot dt\\ i_{ch}\left(i\right)=k_{1}\cdot {\left(v_{GS}\left(i\right)-V_{th}\right)}^{x}+k_{2}\; \end{array}\right.$$

where *i* stands for the *i*th iteration. The trend of $i_{D}$ or $i_{ch}$ during this time is not linear; therefore, a numerical integration method needs to be adopted to evaluate the switching losses: the trapezoidal rule, (11), is employed. The time interval is always dt=0.01 ns. The voltage across the MOSFET during $t_{ri}$ does not change and remains equal to $V_{DC}$.

(11) $$\left\{\begin{array}{c} v_{DS}=V_{DC}\\ \int_{t^{*}}^{t^{*}+dt}V_{DC}\cdot i_{ch}dt=V_{DC}\cdot \frac{[i_{ch}\left(t^{*}\right)+i_{ch}\left(t^{*}+dt\right)]\cdot dt}{2} \end{array}\right.$$

(12) $$E_{on,t_{ri}}=V_{DC}\cdot \sum_{k=0}^{n-1}\frac{[i_{ch}\left(k\right)+i_{ch}\left(k+1\right)]\cdot dt}{2}$$

where n·dt corresponds to the effective rise time. In this way, the non-linear trend of the channel current is considered. The other contribution to the switch-on losses is due to the voltage falling. To estimate the voltage falling time $t_{fu}$, points are extracted from the $\left(C_{rss}-v_{DS}\right)$ curve that is found in the datasheet. A curve fitting could be used, as in [17], but a zero-hold interpolation of the extracted points is simple and effective. The number of points should be significant, and the sampling rate should be higher, near the “knee” of the curve; in this case, 68 $C_{rss}$ values are taken. Since $C_{rss}$ changes significantly, along with $v_{DS}$, a weighted average value is calculated for $t_{fu}$. The following indexes are defined (13):

(13) $$\left\{\begin{array}{c} l=\max_{k}\left\{v_{DS,C_{rss}}\left(k\right)|v_{DS,C_{rss}}\left(k\right)<=V_{DS,on}\right\}\\ h=\max_{k}\left\{v_{DS,C_{rss}}\left(k\right)|v_{DS,C_{rss}}\left(k\right)<=V_{DC}\right\} \end{array}\right.$$

where $v_{DS,C_{rss}}\left(k\right)$ is the $k^{th}$ sample of the reverse-transfer capacitance curve. After defining the extremes, $t_{fu}$ is initialized, considering that $C_{rss}=C_{rss}\left(h\right)$ is discharged at a constant current $I_{G,on}$ (14), and then, $dv_{DS}/dt$ is initialized as well (15).

(14) $$I_{G,on}=\frac{V_{dr}-V_{mil}}{R_{G,on}}$$

(15) $$\left\{\begin{array}{c} t_{fu}=\frac{\left(V_{DC}-V_{DS,on}\right)\cdot C_{rss}\left(h\right)}{I_{G,on}}\\ \frac{dv_{DS}}{dt}=-\frac{V_{DC}-V_{DS,on}}{t_{fu}} \end{array}\right.$$

where $V_{mil}$ assumes the value calculated in (8). At each iteration of the *while* cycle, $v_{DS}$ is updated and a variable *c* is increased by one unit to count the integer number of dt, during which $C_{rss}$ is considered constant to the sample value $C_{rss}\left(k\right)$ (16). Whenever $v_{DS}>v_{DS,C_{rss}}\left(k\right)$, *k* is decreased by one unit and *c* is reset to zero: this operation can occur until k≥l (17). Saving the value of c(k) for each $C_{rss}\left(k\right)$ sample, the weighted average value of $t_{fu}$ can be calculated (18) at the end of the cycle, when $v_{DS}=v_{DS,on}$.

(16) $$\left\{\begin{array}{c} c(k)=c(k)+1\\ v_{DS}\left(i\right)=v_{DS}\left(i-1\right)+\frac{dv_{DS}}{dt}\left(k\right)\cdot dt \end{array}\right.$$

(17) $$\left\{\begin{array}{c} k=k-1\\ c(k)=1\\ t_{fu}\left(k\right)=\frac{\left(V_{DC}-V_{DS,on}\right)\cdot C_{rss}\left(k\right)}{I_{G,on}}\\ \frac{dv_{DS}}{dt}\left(k\right)=-\frac{V_{DC}-V_{DS,on}}{t_{fu}\left(k\right)} \end{array}\right.$$

(18) $$t_{fu}=\frac{\sum_{k=l}^{h}t_{fu}\left(k\right)\cdot c\left(k\right)}{\sum_{k=l}^{h}c\left(k\right)}$$

Assuming the linear falling of the voltage during this time, (19) represents the other contribution to the switch-on losses.

(19) $$E_{on,t_{fu}}=\frac{1}{2}V_{DC}I_{0}\cdot t_{fu}$$

$E_{on}$ becomes the algebraic sum of the two contributes.

(20) $$E_{on}=E_{on,t_{ri}}+E_{on,t_{fu}}$$

#### 2.7.3. $E_{off}$ Calculation

The $E_{off}$ losses also consists of two contributes: $E_{off,t_{ru}}$, due to the non-instantaneous voltage rising, and $E_{off,t_{fi}}$, due to the current falling. The method employed to evaluate $t_{ru}$ is the same as that for $t_{fu}$, but the equations in (14)–(18) must be re-arranged. The charging current for $C_{rss}$ becomes (21) if a zero voltage is applied by the gate driver. *k* is increased until k≤h, and the while cycle stop condition is $v_{DS}=V_{DS,on}$.

(21) $$I_{G,off}=\frac{V_{mil}}{R_{G,off}}$$

(22) $$\left\{\begin{array}{c} t_{ru}=\frac{\left(V_{DC}-V_{DS,on}\right)\cdot C_{rss}\left(l\right)}{I_{G,off}}\\ \frac{dv_{DS}}{dt}=\frac{V_{DC}-V_{DS,on}}{t_{ru}} \end{array}\right.$$

(23) $$\left\{\begin{array}{c} c(k)=c(k)+1\\ v_{DS}\left(i\right)=v_{DS}\left(i-1\right)+\frac{dv_{DS}}{dt}\left(k\right)\cdot dt \end{array}\right.$$

(24) $$\left\{\begin{array}{c} k=k+1\\ c(k)=1\\ t_{ru}\left(k\right)=\frac{\left(V_{DC}-V_{DS,on}\right)\cdot C_{rss}\left(k\right)}{I_{G,off}}\\ \frac{dv_{DS}}{dt}\left(k\right)=\frac{V_{DC}-V_{DS,on}}{t_{ru}\left(k\right)} \end{array}\right.$$

(25) $$t_{ru}=\frac{\sum_{k=l}^{h}t_{ru}\left(k\right)\cdot c\left(k\right)}{\sum_{k=l}^{h}c\left(k\right)}$$

It follows that $E_{on,t_{ru}}$ can be evaluated as in (26).

(26) $$E_{off,t_{ru}}=\frac{1}{2}V_{DC}I_{0}\cdot t_{ru}$$

In addition to $t_{ri}$, $t_{fi}$ varies along with $i_{D}$; therefore, a step-by-step method is used similarly to that for $E_{on,t_{ri}}$. In this case, $V_{mil}$ has already been calculated during the evaluation of $E_{on}$ losses. In (27), the initialization of the variables is shown.

(27) $$\left\{\begin{array}{c} v_{GS}=V_{mil}\\ dv_{GS}/dt=\frac{-V_{mil}+V_{th}}{t_{ri}} \end{array}\right.$$

A while cycle is always considered to continue until $v_{GS}>V_{th}$ becomes false or $i_{ch}$>$I_{0}$ becomes false. Inside the cycle, if the new value of $i_{ch}$ is lower than the mean value between one sample of $i_{D}$ and the following one, $v_{GS}$ and $dv_{GS}/dt$ are updated. Firstly, $t_{fi}$ is updated to the next sampled value, and then, $dv_{GS}/dt$ is evaluated again. At each iteration of the *while* cycle, whether the inequality mentioned above is true or not, the values are updated similarly to that for the switch-on losses (10). Subsequently, $E_{off,t_{ri}}$ has the following expression.

(28) $$E_{off,t_{fi}}=V_{DC}\cdot \sum_{i=0}^{n-1}\frac{[i_{ch}\left(i\right)+i_{ch}\left(i+1\right)]\cdot dt}{2}$$

$E_{off}$ losses are then the sum of two contributions (29):

(29) $$E_{off}=E_{off,t_{fi}}+E_{off,t_{fu}}$$

**Figure 2:**
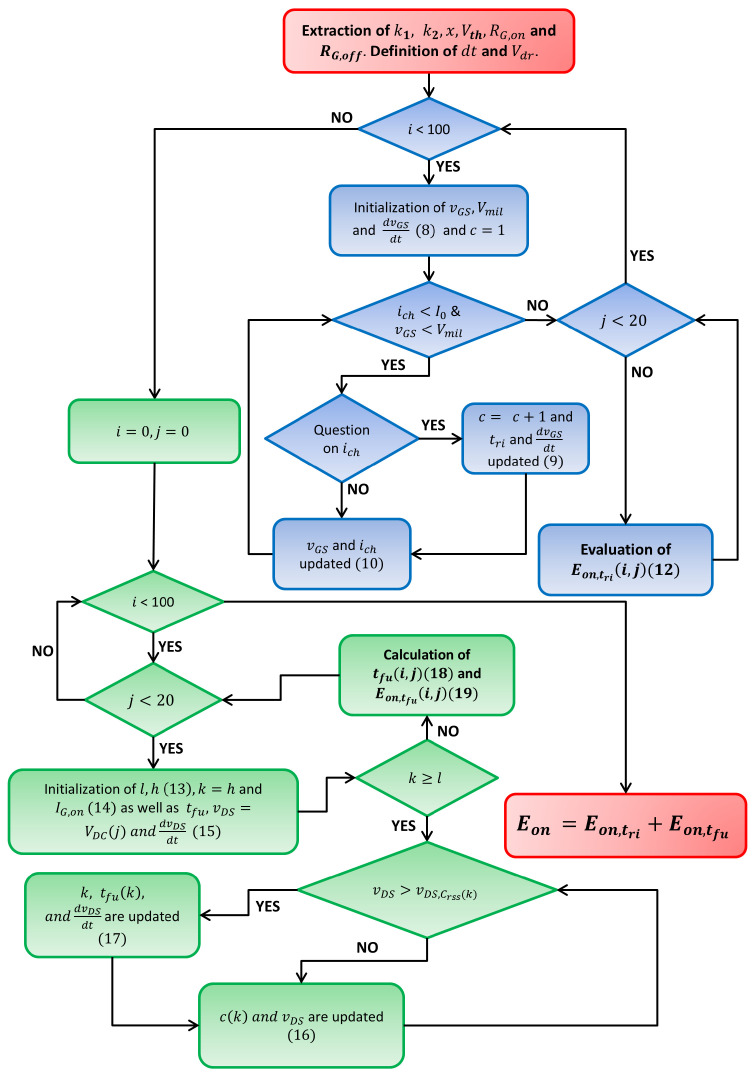
Flow chart of the algorithm used to evaluate $E_{on}$.

**Figure 3:**
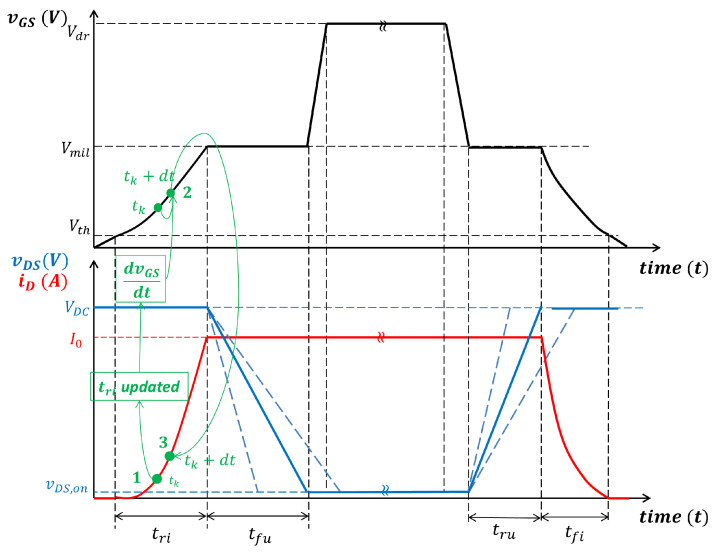
Switching transients assumed for the algorithm implementation.

The algorithm used for the calculation of the losses $E_{on}$ is summarized in Figure 2 and can be implemented in MATLAB R2024a. In this figure, *i* is the index of the current values $I_{0}$ = 0, 0.5 A, 1.0 A, 1.5 A, …, 50 A, and *j* is the index of supply voltage values $V_{DC}$ = 0 V, 10 V, 20 V, …, 100 V, 200 V, …, 1000 V. That is very similar to the one used to evaluate $E_{off}$. Consider that it is implemented for $T_{j}=25$ °C and $T_{j}=150$ °C. In Figure 3, the algorithm is explained graphically and the assumed switching transients are shown for the gate source voltage and the drain source voltage and current. Once the $E_{on}$ and $E_{off}$ matrices have been evaluated, they can be uploaded as a look-up table in PLECS to evaluate power losses due to the switching process in several simulating conditions [29]. The efficiency results over the half-bridge architecture are shown in comparison with the experimental results; see Section 4.

#### 2.7.4. Universality and Further Discussions on the Proposed Model

**Figure 4:**
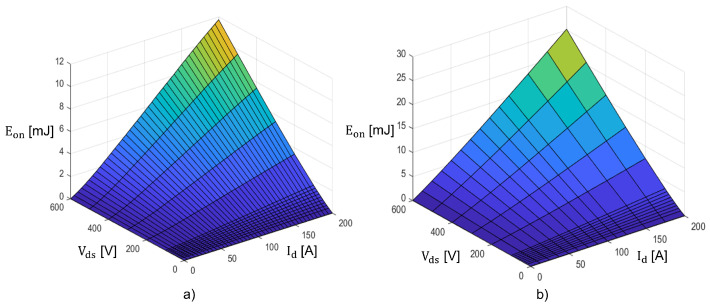
Comparison of the $E_{on}$ losses at $T_{j}=25$ °C between (**a**) IMW65R020M2H and (**b**) IMW65R007M2H.

The proposed model is meant to be applied to any SiC MOSFET since it is not dependent on the individual part number technology: all the equations in the previous subsection do not include any reference to a specific topology. To further underline that, the algorithm was applied to two 2nd Gen. CoolSiC: IMW65R007M2H [30] and IMW65R020M2H [31]. These have the same blocking voltage, technology, and package but very different switching losses and $R_{DSon}$: 7 mΩ and 20 mΩ, respectively. As shown in Figure 4, the algorithm results are in agreement with the information provided by the manufacturer. Since it implies an iterative method, any development environment in C or C++ can be employed as a substitution of MATLAB; therefore, its applicability is also notable. In this paper, an application with all-SiC board, SiC MOSFETs, and SiC Schottky diodes in anti-parallel was employed. If MOSFET body diodes were employed, reverse recovery should be considered: in this case, it is sufficient to add the calculations found in [16] or [17] at the end of the algorithm.

#### 2.7.5. Virtual Junction Temperature Estimation

The estimation of the junction temperature is the first and most important step in evaluating the thermal stress and lifetime of the SiC MOSFET. If $P_{loss}$ is the sum of the switching and conduction losses of a single device under a certain operating condition, it is sufficient to employ the turn-on and turn-off losses as look-up tables in the micro controller to derive the junction temperature $T_{j}$ of the device. The process implicitly requires knowledge of the thermal chain between the junction and the environment. The expression of $T_{j}$ is given in (30).

(30) $$T_{j}\left(t\right)=P_{loss}\left(t\right)\cdot \left[Z_{th,j-h}\left(t\right)+Z_{th,h-a}\left(t\right)\right]+T_{amb}\left(t\right)$$

where $Z_{th,j-h}$ and $Z_{th,h-a}$ are the thermal impedance from junction to heat sink and from heat sink to ambient. The first one is generally expressed as $Z_{th,j-h}\left(t\right)=Z_{th,j-c}\left(t\right)+Z_{th,c-h}\left(t\right)$, but only if the device comes in a package. Otherwise, if a bare-die part number is chosen in the application, the two terms collapse into one. $Z_{th,j-c}$ can be derived from the part number datasheet curve for the evaluation of $Z_{th,c-h}$ or $Z_{th,j-h}$ in the case where a bare-die component [32] can be used. Each thermal impedance can be represented as a foster thermal RC network, thus the explanation of the temperature dependency of these quantities. $P_{loss}$ changes along with the operating condition and $T_{j}$; $T_{amb}$ should be continuously updated; and therefore, they are also time-dependent. If an operating condition is maintained until a stable temperature is reached, only the thermal resistances are significant and the expression in (30) transforms into (31).

(31) $$T_{j}=P_{loss}\cdot \left[R_{th,j-h}+R_{th,h-a}\right]+T_{amb}$$

The switching loss model calculates $E_{on}$ and $E_{off}$ from the load current and the bus voltage; thus, a commercial current sensor like the coreless TLI4971 [33], from Infineon, employed for the board tested in this work can be used. The bus voltage can instead be measured with a simple voltage divider. Since these physical quantities are important for every electric power drive unit or converter connected to the grid, the implementation of the virtual temperature sensor based on the proposed NAM model is straightforward and requires sustainable efforts.

## 3. Experimental Setup

**Figure 5:**
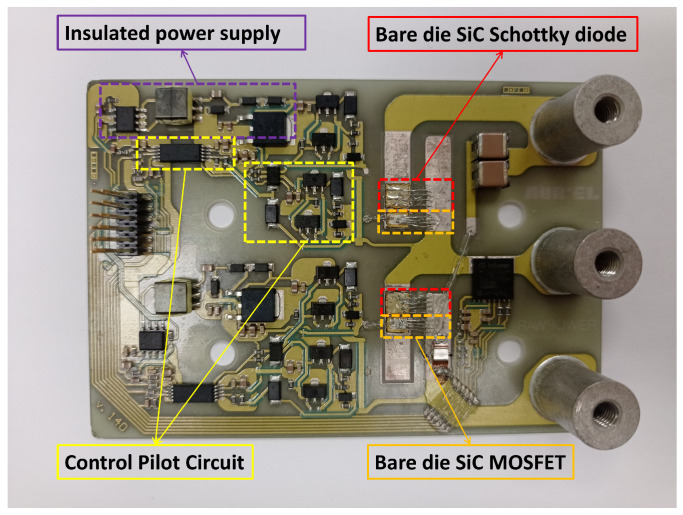
All-SiC half-bridge module.

**Figure 6:**
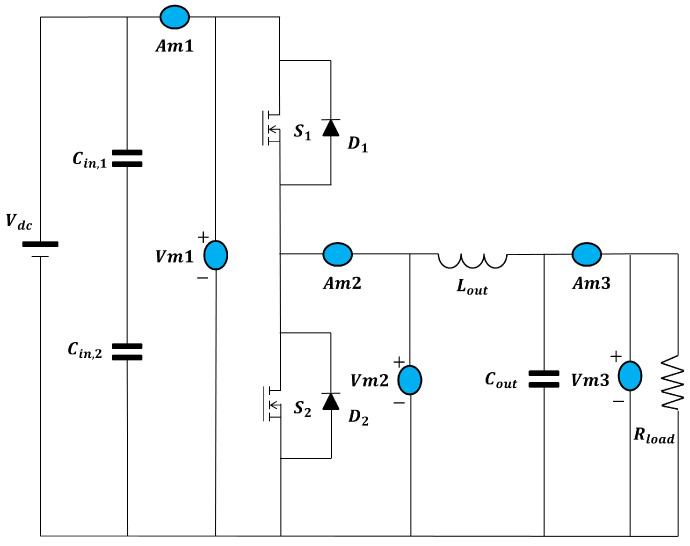
Wiring diagram.

**Figure 7:**
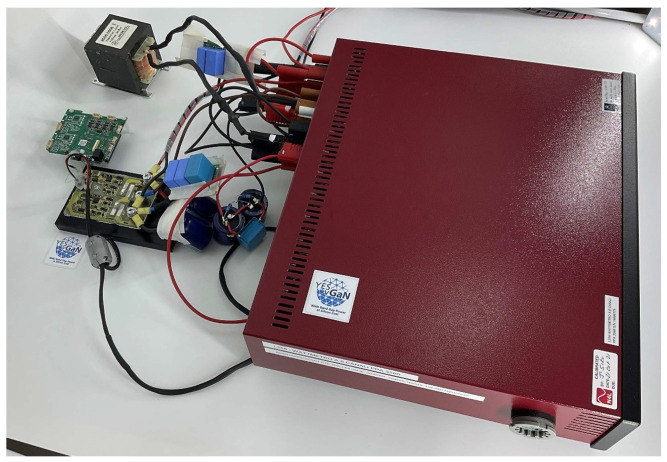
Measurement setup.

The conversion unit has been realized as a PCB, produced over a ceramic support that also contains the insulated power supply for the Control Pilot Circuit. The components are two SiC MOSFETs, s4101 [34], and two SiC Schottky diodes, s6305 [35], placed in anti-parallel; see Figure 5. The PCB has been assembled over an aluminum heat sink. A STM32F765II micro controller by STMicroelectronics, Geneva, Switzerland has been used to pilot the driver and select both the work frequency and duty cycle. The input filter consists of two film capacitors, one of 56 nF and one of 47 nF, directly placed in parallel to the DC supply voltage source. In addition, three film capacitors (two of 56 nF and one of 1.5 µF) are placed in parallel with three electrolytic capacitors of 470 µF between ’+’ and ’−’ of the half-bridge. The DC power supply provides constant voltage to the half-bridge: experiments are carried out at $V_{DC}$ = 400 V, 450 V and 500 V; the LC output filter consists of a ferrite core inductor of 1.2 mH and a group of three capacitors, one ceramic and two electrolytic of 1.5 µF and 470 µF, respectively. The resistive load is the parallel of two 47 Ω resistances. Voltages, currents, and powers are acquired after the bulk input filter at the input and before and after the LC filter at the output with a PPA 3500 power analyzer, manufactured by N4l (Loughborough, UK), in order to measure the converter efficiency before and after the LC output filter: both measures are needed to compute the filter AC losses. The wiring has been made with short cables to enhance impedance matching and reduce noise during acquisition. The wiring schematic is shown in Figure 6. In Figure 7, the entire measurement setup is shown, apart from the resistive load, which consists of two 47 Ω resistances placed in parallel. The results of the experiments take into account the expanded uncertainty, and they are shown in Section 4.

## 4. Comparison with the Experiments

In order to validate the analytical model for switching loss calculation, the measurement uncertainty is evaluated for several operating conditions. The aim of this calculation is to define a proper way to compare simulations and experiments. Specifically, efficiency estimation is considered competent if the simulation result is included between the range defined by the expanded uncertainty around the mean measured efficiency. Therefore, a series of 11 measurements is conducted on the half-bridge module. Each of these measurements consists of 10 samples, where the RMS voltage, the RMS current, the power in the three channels, and the frequency only in the second channel to which the *LC* filter is connected are acquired. This procedure is repeated for the duty cycles 0.2, 0.3, 0.4, 0.5, and 0.7, as well as for three switching frequencies: 40, 60 and 80 kHz. In this way, for each simulation result, there is a respective experimental range. It is believed that a proper estimation of the real unknown value, or the center of the range, is represented by the mean value over the 11 runs of the arithmetic mean above each of the 10 samples. Then, it is possible to express the A-type uncertainty or the repeatability deviation as in (32), following the guideline in [36].

(32) $$u_{A,j,f}=\sqrt{\frac{\sum_{r=1}^{n}{\left({\bar{\bar{x}}}_{\eta ,j,f}-{\bar{x}}_{\eta ,r,j,f}\right)}^{2}}{n-1}}$$

where *n* equals 11, the number of runs; ${\bar{x}}_{\eta ,r,j,f}$ is the mean efficiency among the 10 samples of the $r^{th}$ run; *j* is the duty cycle index; *f* is the switching frequency index; and ${\bar{\bar{x}}}_{\eta ,j,f}$ is the mean value of the efficiency over the 11 sets of measurements for a specific duty cycle and switching frequency. The computation of the *B-type uncertainty*, instead, depends on the measurement instrument. Since the PPA 3500 power analyzer is used for the measurements, the authors refer to the user manual [37] to define this bias. For both input and output power, the accuracy of the power measurement is expressed by (33).

(33) $$u_{max,W_{r}}=\frac{\left(0.1+\frac{0.1}{PF}+\frac{0.01\cdot f_{sw}}{PF}\right)\cdot W_{r}+0.05\cdot V_{p}I_{p}}{100}$$

where $V_{p}$ and $I_{p}$ are the voltage and ampere range of the instruments and $W_{r}$ is the measured power, which can be either substituted with $W_{in}$ or $W_{out}$. $u_{max,W_{r}}$ represents the maximum deviation in [W] from the mean measured power. PF is the power factor, which can be simply evaluated by dividing the measured power by the product of the input voltage and the input current, and $f_{sw}$ is the switching frequency expressed in kHz, 40, 60, or 80. Once the maximum deviations, $u_{max,W_{out}}$ and $u_{max,W_{in}}$, are found, they are then put together to find the B-type uncertainty for the measured output efficiency; see (34). The reason for $\sqrt{3}$ is the assumption of a uniform distribution of the systematic error.

(34) $$u_{B,j,f}=\sqrt{{\left(\frac{u_{max,W_{in,j,f}}}{V_{p}I_{p}\sqrt{3}}\right)}^{2}+{\left(\frac{u_{max,W_{out,j,f}}}{V_{p}I_{p}\sqrt{3}}\right)}^{2}}\cdot 100$$

The compound uncertainty is considered the square root of the two components squared (35). Finally, the expanded uncertainty can be defined with a level of confidence of 95%, therefore with a coverage factor $k_{p}=2$; see (36).

Applying these formulae, it is possible to find range values around the mean efficiency for every duty cycle and switching frequency, which allows for comparing the simulation results with those of the measurements tests.

(35) $$u_{comp,j,f}=\sqrt{u_{A,j,f}^{2}+u_{B,j,f}^{2}}$$

(36) $$u_{k,j,f}=k_{p}u_{comp,j,f}$$

The uncertainty range is defined in (37), and it varies under different test conditions.

(37) $$\left[{\bar{\bar{x}}}_{\eta ,j,f}-u_{k,j,f};{\bar{\bar{x}}}_{\eta ,j,f}+u_{k,j,f}\right]$$

As shown in Table 2, Table 3 and Table 4, the simulation results are consistent with the experimental tests. The efficiencies estimated throughout PLECS show great compliance with the measurements at 40, 60, and 80 kHz. To compare this work with the state of the art, the authors investigated three additional models. The first is the old simplified Infineon model [9]; the second is a NAM model developed by Christen and Biela [16]; and the last is a FAM model, initially developed in [23] and then refined in [24] by Hu and Biela. These, similarly to the one presented in this work, are based on datasheet parameters. As stated at the beginning of this work, other authors introduced further levels of sophistication at the expense of carrying out some preliminary measurements. Since the proposed model is entirely based on the information provided by the manufacturers, a comparison with those would be beyond the scope of this paper. To develop the reference models, first, a MATLAB script was built; then, the resultant losses were uploaded in the thermal description of the device in PLECS; and as a last step, simulations were carried out. Except for the algorithm, the same procedure employed in this work was also applied to the state-of-the-art models. To make a comparison between the proposed model and the existing ones, four KPIs were used. Each KPI should be intended per switching frequency $f_{sw}$ (Table 2, Table 3 and Table 4).

**Figure 8:**
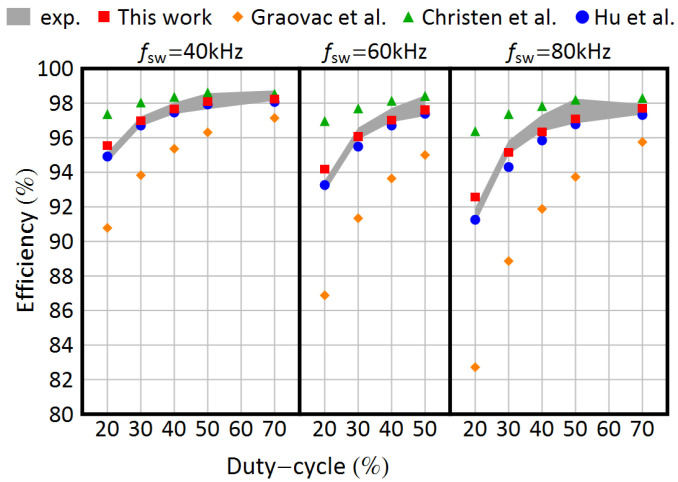
Comparison among experimental and simulation results. The filled area represents the experimental results, including measurement uncertainty. Simulation results are reported for the model proposed in this work (filled rectangle) and those proposed in [9] (filled diamond), in [16] (filled triangle) and in [24] (filled circle).

- The number of simulation results $n^{\circ }$, which lie in the uncertainty range defined around the measured efficiency.
- The mean absolute error $\bar{e}$, which represents the average displacement with respect to the measured value (38).
- The mean absolute percentage error ${\bar{e}}_{\%}$, which is the unsigned average displacement normalized over the measured value (39). This error suggests the average displacement with respect to the test itself.
- The Modified Mean Absolute Percentage Error MMAPE (40) is a well-known relative measure of the forecasting accuracy [38].

(38) $$\bar{e}=\sum_{d}\frac{|\eta_{s,d,f}-{\bar{\bar{x}}}_{\eta ,d,f}|}{N_{f}}$$

(39) $${\bar{e}}_{\%}=\frac{1}{N_{f}}\cdot \sum_{d}∥\frac{\eta_{s,d,f}-{\bar{\bar{x}}}_{\eta ,d,f}}{{\bar{\bar{x}}}_{\eta ,d,f}}∥\cdot 100\%$$

(40) $$MMAPE=\frac{1}{N_{f}}\cdot \sum_{d}∥\frac{\eta_{s,d,f}-{\bar{\bar{x}}}_{\eta ,d,f}}{\eta_{s,d,f}+{\bar{\bar{x}}}_{\eta ,d,f}}∥$$

where $\eta_{s,d,f}$ is the estimated efficiency at a certain duty *d*, ${\bar{x}}_{\eta ,d,f}$ is the mean measured efficiency, and $N_{f}$ represents the number of cases for a frequency *f*. At 40 kHz, the deviation between the proposed model and the experimental test is significant only for a duty d=0.2, which means at low currents, the remaining five values show great compliance with the experimental tests. The same happens at 60 and 80 kHz: three simulation results fall into the uncertainty range in both cases, and the displacement is only relevant at low currents. The estimated efficiencies that fall into the uncertainty range are highlighted in bold in Table 2, Table 3 and Table 4. The proposed model appears to be superior to the ones chosen for the comparison by each KPI: the simplified model significantly overestimates the switching losses, but instead, Christen’s model [16] underestimates them; the authors believe that it is mainly due to the equivalent $C_{GD}$ capacitance approximation. Hu and Biela’s model in [24] also shows good compliance with the experimental tests, even if the proposed one still wins over in terms of KPIs and displacement with respect to the measured efficiency, excluding the low-current-condition case. The efforts made by Hu and Biela in developing a low computation time and yet accurate model is indisputable, but as the authors themselves claim in the paper, the mathematics are very challenging and also the algorithm is quite complicated. Furthermore, in favor of the computational time, some initial and strong assumptions are made, such as the step variation of the capacitances $C_{GD}$, $C_{iss}$, and $C_{DS}$; the linear step-wise interpolation of the transfer characteristic; and the temperature independency of the switching losses. In fact, not every manufacturer provides the variation in switching losses with the junction temperature $T_{j}$. These simplifications lead to a deviation of the model from the measured values. The deviation is more and more prominent as the switching frequency increases (see Table 4), and the reason is trivial: the error on the energy losses increases linearly with the switching frequency. Especially for high-power applications, this deviation can be relevant for the thermal, heat sink design. In Figure 8, the simulation results are summarized and shown along with the uncertainty range.

## 5. Conclusions

SiC devices enable higher efficiency and power density due to faster switching and lower conduction losses, as well as operation at elevated temperatures. A comprehensive loss model supports the development of online virtual sensors for indirect efficiency and junction temperature monitoring. As power density increases, thermal stress on MOSFETs grows, which without proper loss and device status monitoring, reduces system MTTF. This paper proposes an effective analytical model to estimate SiC MOSFET switching losses, based exclusively on information derived from datasheets. The model is kept as simple as possible by neglecting some secondary effects on total switching losses, such as the charging and discharging of the output capacitance $C_{oss}$, which is a useful approximation and does not hinder its applicability in the power electronics industry. Additional assumptions regarding parasitic inductances and the short-channel effect are made to avoid preliminary and time-consuming measurements for parameter extraction. The reverse-recovery approximation applies specifically to the case studied in this work, although this contribution can be easily integrated if needed. Despite these simplifications, the model proved to be consistent with experimental tests and outperformed state-of-the-art models across all selected KPIs. A maximum MMAPE of 0.51 % was recorded at 80 kHz. The accuracy of the model is validated by the experimental results presented in Section 4, which also account for measurement uncertainty. The model demonstrates high versatility, as it can be applied to any SiC MOSFET part number for all-SiC applications and can be easily adapted to lower-performance drives without SiC Schottky diodes. Since the algorithm can run in both C/C++ environments and MATLAB scripts, its implementation for a virtual sensor or online efficiency measurements is also straightforward.

## 6. Future Developments

As a further development, the authors aim to implement the model for the calculation of efficiency and temperature in real time under various load conditions in a typical electric drive application. These tests will also prove the effectiveness of the proposed model in estimating junction temperature and allow for a proper lifetime evaluation, which will be carried out in the next work.

## Figures and Tables

**Table 1:** Overview of the state-of-the-art switching loss models.

Model	Advantages	Drawbacks
[9]	Very simple to implement	Very inaccurate
[10]	Formally complete	Discrete accuracy and preliminary tests
[11,12]	Accurate	Dynamic MOSFET characterization
[13]	Detailed	Lack of $E_{off}$ losses
[14,15,17]	Accurate and complete	Complex and preliminary tests
[18,19]	Specific	Dynamic $C_{GD}$ extraction
[16,20,21,22]	Based on datasheet parameters	$g_{m}$ and $C_{GD}$ approximation, discrete accuracy
[23,24]	Accurate and based on datasheet parameters	Extremely complex, step approximation of the capacitance and temperature independency

**Table 2:** Efficiency comparisons at 40 kHz.

$V_{\mathrm{DC}}$ [V]	Duty [p.u]	Simplified Model [9] $\eta_{s}$ [%]	Model in [16] $\eta_{s}$ [%]	Model in [24] $\eta_{s}$ [%]	Proposed Model $\eta_{s}$ [%]	$\left[{\bar{\bar{x}}}_{\eta }-u_{k}\right]$	Measured ${\bar{\bar{x}}}_{\eta }$ [%]	$\left[{\bar{\bar{x}}}_{\eta }+u_{k}\right]$
400	0.2	90.78	97.37	**94.93**	95.54	94.57	94.87	95.16
400	0.3	93.83	98.04	**96.70**	**96.97**	96.66	96.95	97.23
400	0.4	95.36	98.37	**97.46**	**97.65**	97.37	97.7	98.03
400	0.5	96.31	98.62	**97.94**	**98.09**	97.65	98.12	98.58
400	0.7	97.14	**98.54**	98.07	**98.23**	98.14	98.44	98.74
450	0.7	96.81	98.33	**97.81**	**98.01**	97.80	98.04	98.28
500	0.3	93.35	97.73	96.60	**96.83**	96.80	97.02	97.24
$n^{\circ }$		0	1	5	6			
$\bar{e}$		2.51	0.84	0.25	0.17			
${\bar{e}}_{\%}$		2.59	0.87	0.26	0.18			
**MMAPE**		2.63	0.86	0.26	0.18			

In bold, the simulation results which lie in the uncertainty range.

**Table 3:** Efficiency comparisons at 60 kHz.

$V_{\mathrm{DC}}$ [V]	Duty [p.u]	Simplified Model [9] $\eta_{s}$ [%]	Model in [16] $\eta_{s}$ [%]	Model in [24] $\eta_{s}$ [%]	Proposed Model $\eta_{s}$ [%]	$\left[{\bar{\bar{x}}}_{\eta }-u_{k}\right]$	Measured ${\bar{\bar{x}}}_{\eta }$ [%]	$\left[{\bar{\bar{x}}}_{\eta }+u_{k}\right]$
400	0.2	86.88	96.97	**93.25**	94.17	92.84	93.18	93.53
400	0.3	91.34	97.70	95.5	**96.06**	95.89	96.23	96.58
400	0.4	93.64	98.14	96.71	**97.02**	96.9	97.31	97.72
400	0.5	95.00	**98.42**	**97.38**	**97.60**	97.25	97.85	98.45
$n^{\circ }$		0	1	2	3			
$\bar{e}$		4.43	1.67	0.47	0.43			
${\bar{e}}_{\%}$		4.63	1.76	0.48	0.45			
**MMAPE**		4.75	1.73	0.48	0.45			

In bold, the simulation results which lie in the uncertainty range.

**Table 4:** Efficiency Comparisons at 80 kHz.

$V_{\mathrm{DC}}$ [V]	Duty [p.u]	Simplified Model [9] $\eta_{s}$ [%]	Model in [16] $\eta_{s}$ [%]	Model in [24] $\eta_{s}$ [%]	Proposed Model $\eta_{s}$ [%]	$\left[{\bar{\bar{x}}}_{\eta }-u_{k}\right]$	Measured ${\bar{\bar{x}}}_{\eta }$ [%]	$\left[{\bar{\bar{x}}}_{\eta }+u_{k}\right]$
400	0.2	82.72	96.38	**91.26**	92.57	91.03	91.48	91.94
400	0.3	88.86	97.37	94.32	**95.14**	95.03	95.44	95.84
400	0.4	91.88	97.84	95.86	96.32	96.36	96.85	97.33
400	0.5	93.73	**98.2**	96.79	**97.09**	96.83	97.54	98.26
400	0.7	95.75	98.30	97.33	**97.7**	97.34	97.65	97.96
$n^{\circ }$		0	1	1	3			
$\bar{e}$		5.20	1.83	0.68	0.48			
${\bar{e}}_{\%}$		5.49	1.95	0.71	0.51			
**MMAPE**		5.68	1.92	0.71	0.51			

In bold, the simulation results which lie in the uncertainty range.

## Data Availability

All data and additional information about the experimental setup and the code can be provided under requirement.

## Abbreviations

The following abbreviations are used in this manuscript:

NAM	Num-analytical model
FAM	Full analytical model
$C_{DS}$	Drain source capacitance
$C_{GD}$	Gate drain capacitance
$C_{GS}$	Gate source capacitance
$C_{iss}$	Input capacitance
$C_{oss}$	Output capacitance
$C_{oss,eq}$	Equivalent output capacitance
dt	Algorithm discrete time-step
$E_{off}$	Turn-off losses
$E_{off,tfi}$	Falling current contribution to the $E_{off}$ losses
$E_{off,tru}$	Rising voltage contribution to the $E_{off}$ losses
$E_{on}$	Turn-on losses
$E_{on,tri}$	Rising current contribution to the $E_{on}$ losses
$E_{on,tfu}$	Falling voltage contribution to the $E_{on}$ losses
$f_{sw}$	MOSFET switching frequency
$g_{m}$	MOSFET channel transconductance
$I_{0}$	Load current
$i_{ch}$	MOSFET channel current
$i_{D}$	Drain current
$I_{G,on}$	Gate turn-on current during Miller’s plateau
$I_{G,off}$	Gate turn-off current during Miller’s plateau
$I_{oss}$	Current through the output capacitance
$I_{rr}$	Reverse-recovery current
$L_{d}$	Drain parasitic inductance
$L_{s}$	Source parasitic inductance
$Q_{oss,eq}$	Equivalent output charge capacitance
$R_{G,on}$	Turn-on gate resistance
$R_{G,off}$	Turn-off gate resistance
$R_{DS,on}$	MOSFET on-conduction resistance
SiC	Silicon carbide
$t_{f}i$	Current fall time
$t_{f}u$	Voltage fall time
$T_{j}$	Junction temperature
$t_{r}i$	Current rise time
$t_{r}u$	Voltage rise time
$V_{DC}$	DC supply voltage
$V_{Dr}$	Gate driver supply voltage
$v_{DS}$	Drain source voltage
$V_{DS,on}$	MOSFET drain source voltage drop during conduction
$V_{mil}$	Miller’s plateau voltage
$V_{th}$	MOSFET threshold voltage
$u_{k}$	Expanded uncertainty
$\eta_{s}$	Estimated efficiency
${\bar{\bar{x}}}_{\eta }$	Mean measured efficiency
